# Author Correction: High-resolution cryo-EM structure of urease from the pathogen *Yersinia enterocolitica*

**DOI:** 10.1038/s41467-020-19845-z

**Published:** 2020-11-12

**Authors:** Ricardo D. Righetto, Leonie Anton, Ricardo Adaixo, Roman P. Jakob, Jasenko Zivanov, Mohamed-Ali Mahi, Philippe Ringler, Torsten Schwede, Timm Maier, Henning Stahlberg

**Affiliations:** 1 Center for Cellular Imaging and NanoAnalytics, Biozentrum, University of Basel, Mattenstrasse 26, CH-4058 Basel, Switzerland; 2 Biozentrum, University of Basel, Klingelbergstrasse 50/70, CH-4056 Basel, Switzerland; 3 SIB Swiss Institute of Bioinformatics, Biozentrum, University of Basel, Klingelbergstrasse 50/70, CH-4056 Basel, Switzerland

**Keywords:** Molecular evolution, Antimicrobial resistance, Molecular biology, Cryoelectron microscopy, Cryoelectron microscopy

Correction to: *Nature Communications* 10.1038/s41467-020-18870-2, published online 9 October 2020.

The original version of this Article contained an error in Fig. 3, in which panel 3a showed the structures of the ureases from *Y. enterocolitica*, *Y. enterocolitica* and *K. aerogenes* superimposed. In the correct version, panel 3a shows the structure of the *Y. enterocolitica* urease only. This has been corrected in both the PDF and HTML versions of the Article.

