# Supplementary material for: High-resolution cryo-EM structure of urease from the pathogen Yersinia enterocolitica
Source: Nat Commun. 2020 Oct 9;11:5101. doi: 10.1038/s41467-020-18870-2 (PMC7547064; doi:10.1038/s41467-020-18870-2)
Supplement: Supplementary file 1 — Supplementary Information [file 41467_2020_18870_MOESM1_ESM.pdf]

Supplementary Information

High-resolution cryo-EM structure of urease from the pathogen *Yersinia enterocolitica*

Ricardo D. Righetto[1*], Leonie Anton[2*], Ricardo Adaixo[1*], Roman P. Jakob[2], Jasenko Zivanov[1], Mohamed-Ali Mahi[2,3], Philippe Ringler[1], Torsten Schwede[2,3], Timm Maier[2†] and Henning Stahlberg[1†]

[1] Center for Cellular Imaging and NanoAnalytics, Biozentrum, University of Basel, Mattenstrasse 26, CH-4058 Basel, Switzerland.

[2] Biozentrum, University of Basel, Klingelbergstrasse 50/70, CH-4056 Basel, Switzerland.

[3] SIB Swiss Institute of Bioinformatics, Biozentrum, University of Basel, Klingelbergstrasse 50/70, CH-4056 Basel, Switzerland.

[*] *these authors contributed equally to this work.*

† Corresponding authors:

Timm Maier
Biozentrum, University of Basel
Klingelbergstrasse 50/70
CH-4056 Basel, Switzerland
Phone: +41 61 207 21 76
E-mail: timm.maier@unibas.ch

Henning Stahlberg
Center for Cellular Imaging and NanoAnalytics (C-CINA)
Biozentrum, University of Basel
WRO-1058, Mattenstrasse 26
CH-4058 Basel, Switzerland
Phone: +41 61 387 32 62
E-mail: henning.stahlberg@unibas.ch

Supplementary Figures

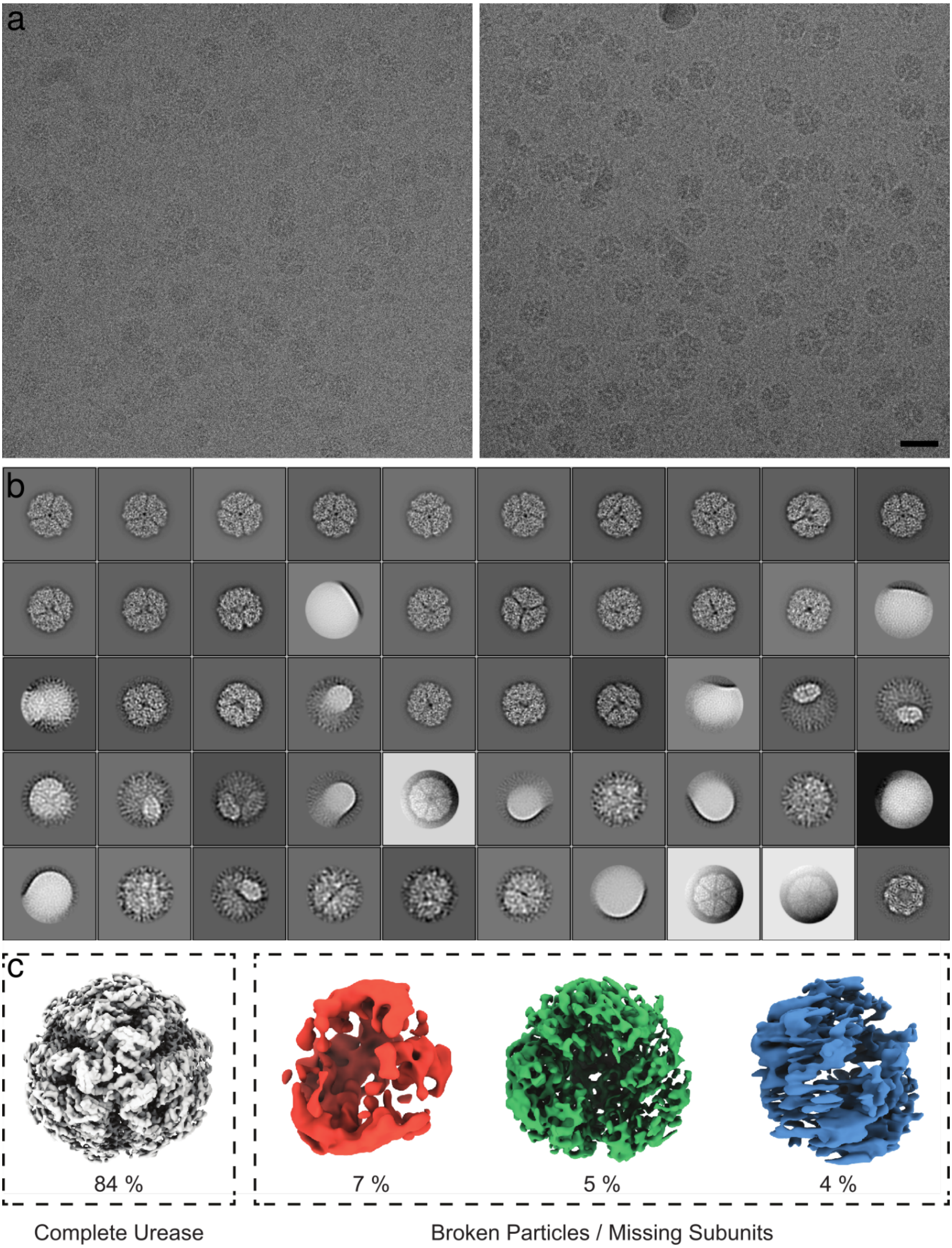

Supplementary Figure 1 Cryo-EM data of *Y. enterocolitica* urease. **a)** Two representative micrographs from the dataset, acquired at -0.64 and -1.36 μm defocus, respectively. Scale bar: 200 Å. **b)** 2D class averages obtained from 194,603 particles in the merged dataset. These averages were obtained with RELION's "Ignore CTF until first peak" option enabled and are sorted by decreasing order of number of particles in each class. Views of urease with missing subunits are observed. The bottom right average shows a contamination by GroEL. **c)** 3D class averages obtained from 141,069 particles in the merged dataset. These averages were obtained without symmetry imposition in RELION. The 3D classes are sorted by the indicated fraction of particles assigned to it. The first class is a complete dodecameric urease assembly, while the other classes represent urease structures with at least one trimer missing from the tetrahedron.

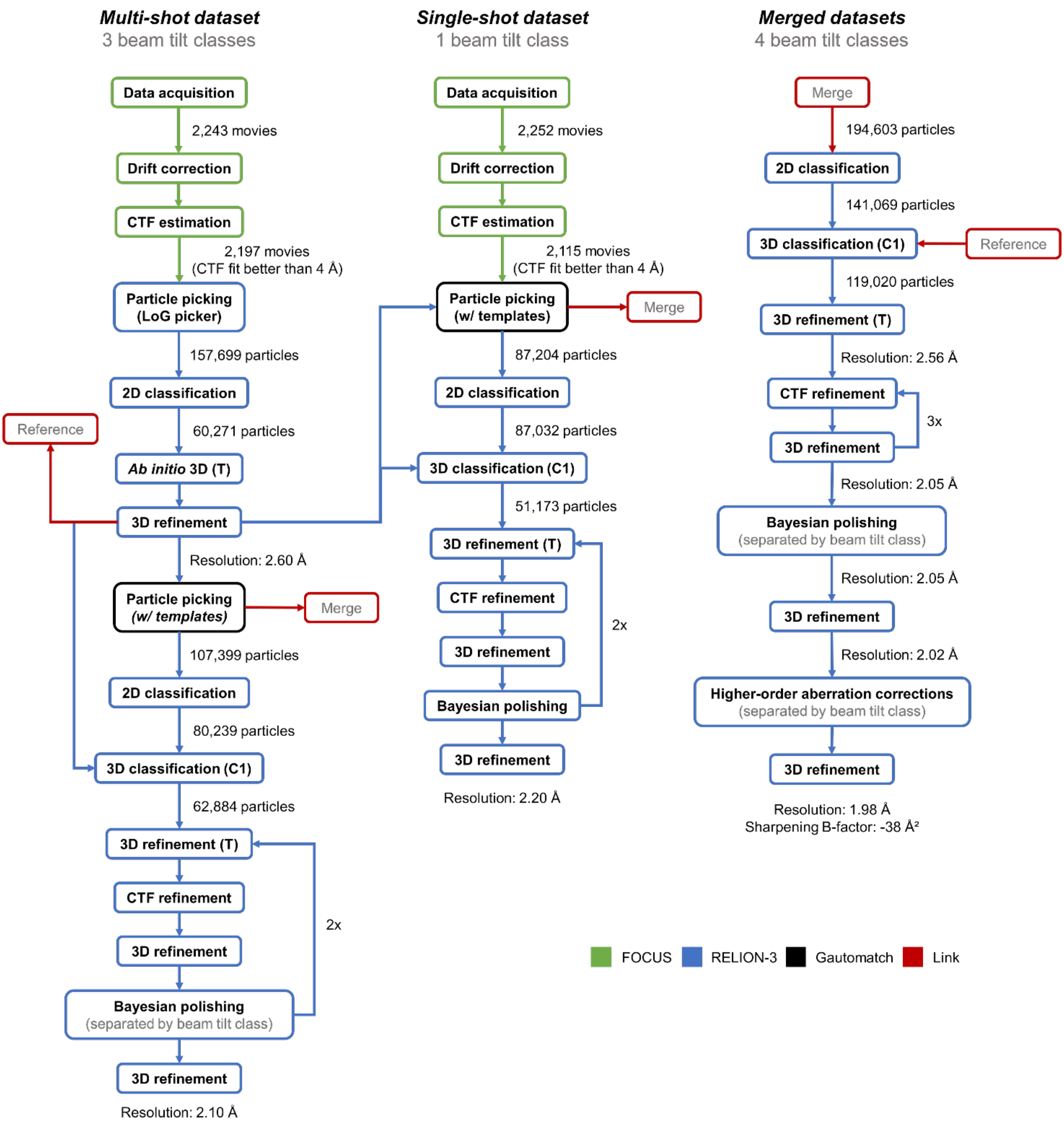

Supplementary Figure 2 Data processing flowchart for the urease cryo-EM map. Masking and postprocessing jobs have been omitted for clarity. All CTF refinement jobs included beam tilt and per-particle defocus refinement (see **Methods** for details). All resolution estimates given correspond to the corrected FSC curves between masked half-maps obtained from postprocessing jobs in RELION [1]. The sharpening B-factor was estimated from the Guinier plot after postprocessing in RELION [2].

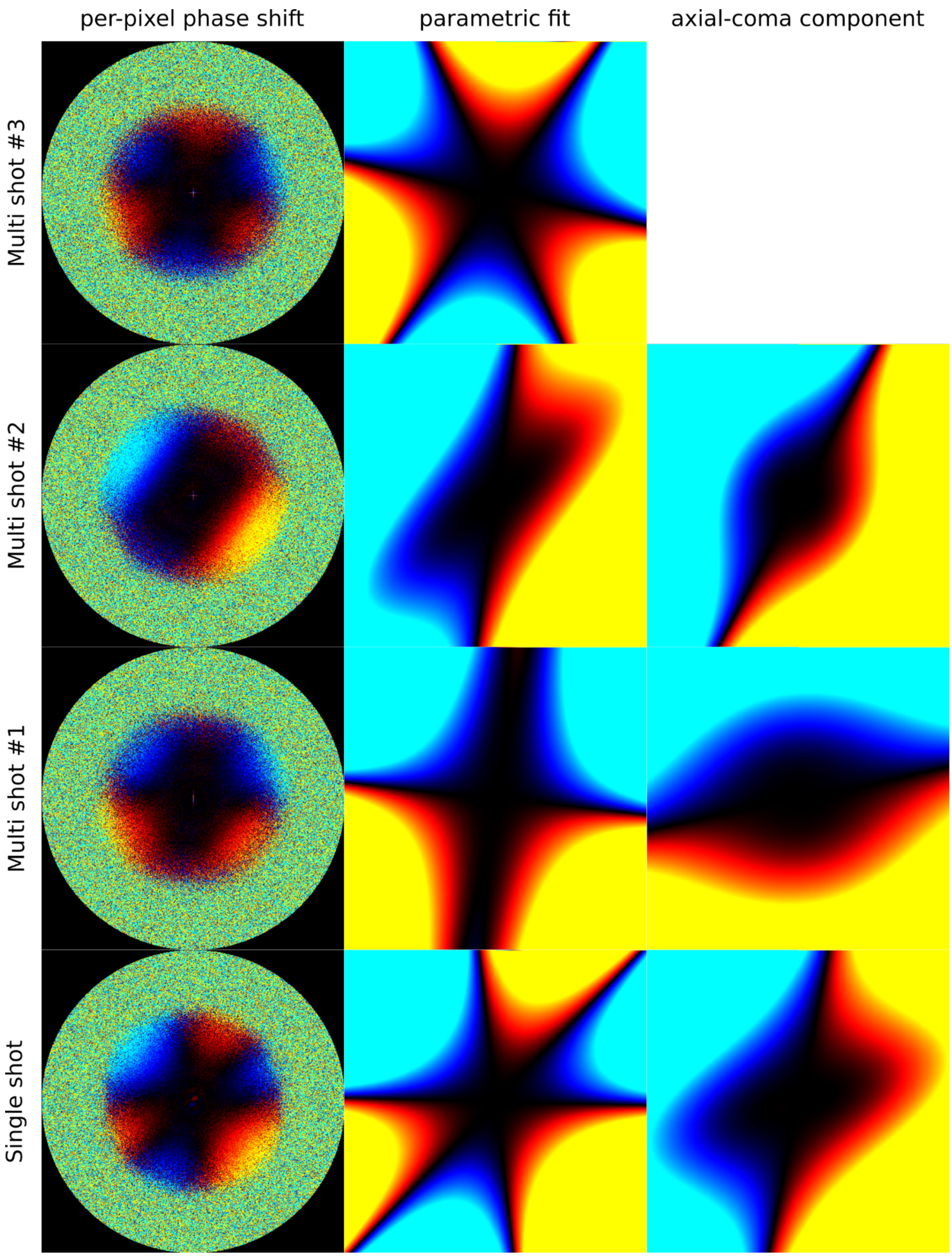
per-pixel phase shift
parametric fit
axial-coma component
Multi shot #3
Multi shot #2
Multi shot #1
Single shot

Supplementary Figure 3 Fits of the anti-symmetrical aberrations arising at the four different beam-shift positions. The left column shows phase shifts measured independently for each Fourier pixel, while the center column shows their parametric fits using third-order Zernike polynomials. The first position (top row, the third beam-shifted multi-shot) corresponds to an essentially untilted beam **(Supp. Tab. 2)**, while the other two multishots and the single-shot dataset exhibit tilts to different extents. Note that even the untilted dataset shows a significant trefoil aberration. In the right column, the parametric fit of the first position has been subtracted, yielding residuals roughly consistent with the axial coma produced by a tilted beam.

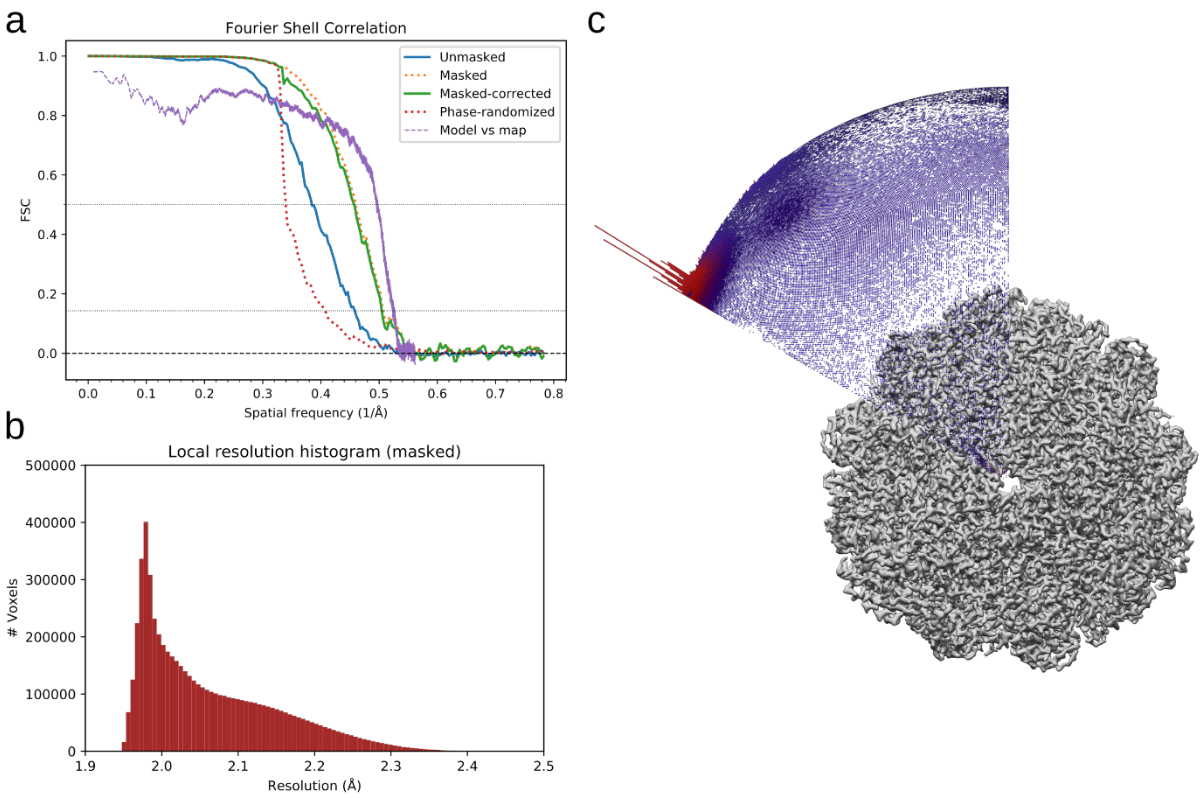

Supplementary Figure 4 Resolution estimates of the urease cryo-EM map. **a)** FSC curves between the half-maps when unmasked (solid blue line), masked (dashed orange), corrected by high-resolution noise substitution after masking (solid green), phase-randomized (dashed red) and between the atomic model and the full experimental map (dashed violet). **b)** Histogram of local resolution assigned to each voxel. **c)** Angular distribution of particles in the urease cryo-EM reconstruction overlaid on the unsharpened map.

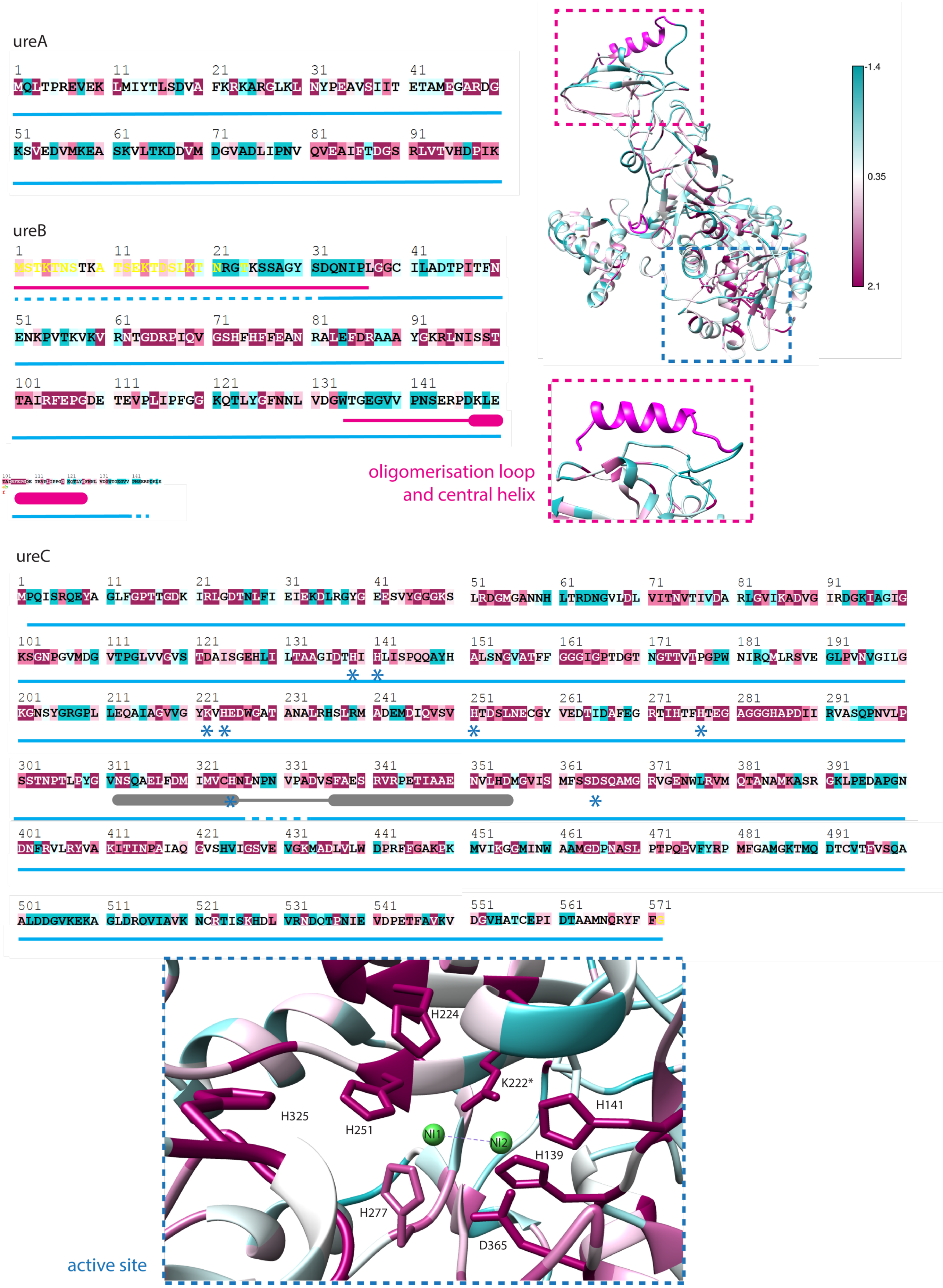
ureA
1 MQLTPREVEK 11 LMIYTLSDVA 21 FKRKARGLKL 31 NYPEAVSIIT 41 ETAMEGARDG
51 KSVEDVMKEA 61 SKVLTKDDVM 71 DGVADLIPNV 81 QVEAIETDGS 91 RLVTVHDPIK
ureB
1 MSTKTNSTKA 11 TSEKTDSLKT 21 NRGTKSSAGY 31 SDQNIPLGGC 41 ILADTPITFN
51 ENKPVTKVKV 61 RNTGDRPIQV 71 GSHFHFFEAN 81 RALEFDRAAA 91 YGKRLNISST
101 TAIRFEPGDE 111 TEVPLIPFGG 121 KQTLYGFNNL 131 VDGWTGEGVV 141 PNSERPDKLE
oligomerisation loop and central helix
-1.4
0.35
2.1
ureC
1 MPQISRQEYA 11 GLFGPTTGDK 21 IRLGDTNLFI 31 EIEKDLRGYG 41 EESVYGGGKS 51 LRDGMGANNH 61 LTRDNGVLDL 71 VITNVTIVDA 81 RLGVIKADVG 91 IRDGKIAGIG
101 KSGNPGVMDG 111 VTPGLVVGVS 121 TDAISGEHLI 131 LTAAGIDTHI 141 HLISPQQAYH 151 ALSNGVATFF 161 GGGIGPTDGT 171 NGTTVTPGPW 181 NIRQMLRSVE 191 GLPVNVGILG
201 KGNSYGRGPL 211 LEQAIAGVVG 221 YKVHEDWGAT 231 ANALRHSLRM 241 ADEMDIQVSV 251 HTDSLNECGY 261 VEDTIDAFEG 271 RTIHTFHTEG 281 AGGGHAPDII 291 RVASQPNVLP
301 SSTNPTLPYG 311 VNSQAEIFDM 321 IMVCHNLNPN 331 VPADVSFAES 341 RVRPETIAAE 351 NVLHDMGVIS 361 MFSSDSQAMG 371 RVGENWLRVM 381 QTANAMKASR 391 GKLPEDAPGN
401 DNFRVLRYVA 411 KITINPAIAQ 421 GVSHVIGSVE 431 VGKMADLVLW 441 DPRFFGAKPK 451 MVIKGGMINW 461 AAMGDPNASL 471 PTPQPVFYRP 481 MFGAMGKTMQ 491 DTCVTFVSQA
501 ALDDGVKEKA 511 GLDRQVIAVK 521 NCRTISKHDL 531 VRNDQTPNIE 541 VDPETFAVKV 551 DGVHATCEPI 561 DTAAMNQRYF 571 F
H224
K222*
H141
H325
H251
NI1
NI2
H139
H277
D365
active site

Supplementary Figure 5 Alignment of 150 ureA, ureB and ureC sequences, chosen by sampling from all homologs found for each protein. ClustalW was used for sequence alignment and the ConSurf server for conservation analysis. Conservation is shown on a gradient from dark purple to white to turquoise (arbitrary units). The light blue bar indicates model completeness. Regions of interest are highlighted: The N-terminal extension in dark blue, the oligomerization loop and its following helix are indicated in magenta, the mobile flap is indicated in grey and the blue asterix indicates residues belonging to the active site.

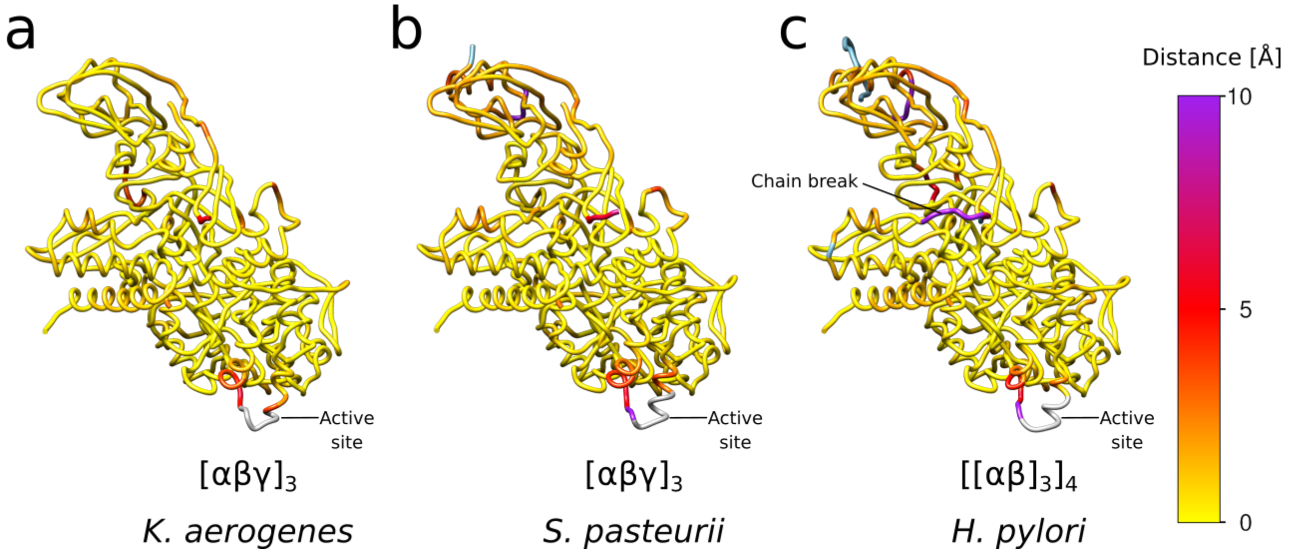

Supplementary Figure 6 Distance comparison between residues of *Y. enterocolitica* urease against ureases with different modes of assembly. Tubes are colored by the pairwise distance of Cα atoms to the corresponding residue in *Y. enterocolitica* urease. Residues without equivalence in *Y. enterocolitica* urease after sequence alignment are shown in blue, while stretches corresponding to residues that have not been resolved in the active site of the cryo-EM structure are shown in gray. Segments with particularly high deviations are indicated.

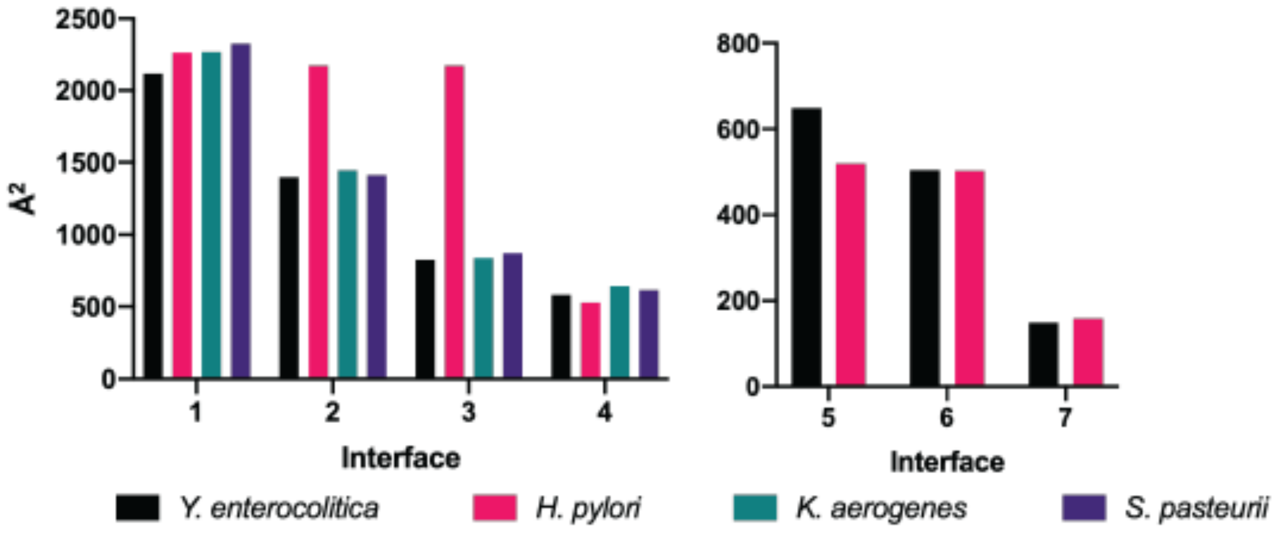

Supplementary Figure 7 Area (Å²) of interfaces 1-7 (numbering as in Figure 4) plotted by organism. Left: intra-trimer interfaces; right: inter-trimer interfaces. The intra-trimer areas of interfaces 2 and 3 in *H. pylori* are higher because they are part of the same chain.

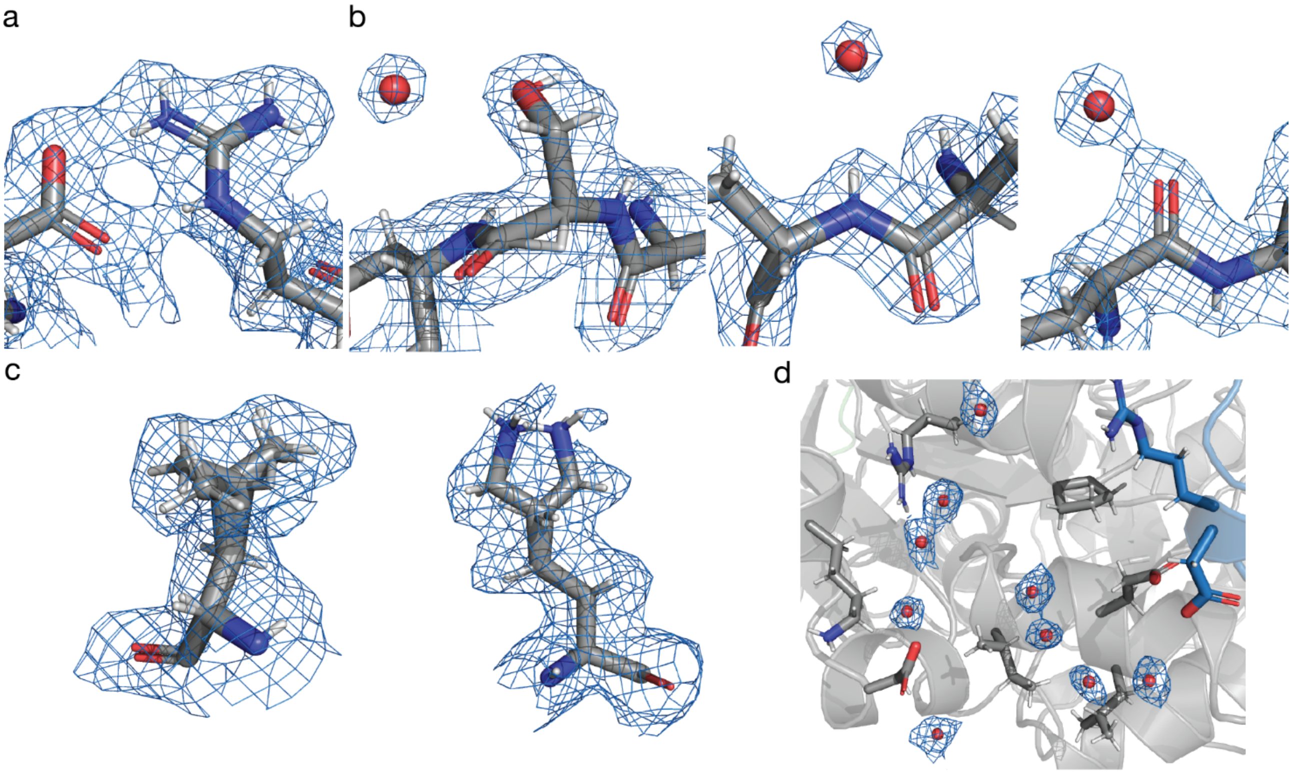

Supplementary Figure 8 High-resolution structural features can be readily seen in the cryo-EM map. **a)** Salt bridge between D69 and R63. **b)** Side chain hydration of S17 and back bone hydration of the oxygen and the nitrogen, respectively. **c)** alternative side chain conformations of L406 and K2. d) network of waters between two ureC proteins (grey, blue).

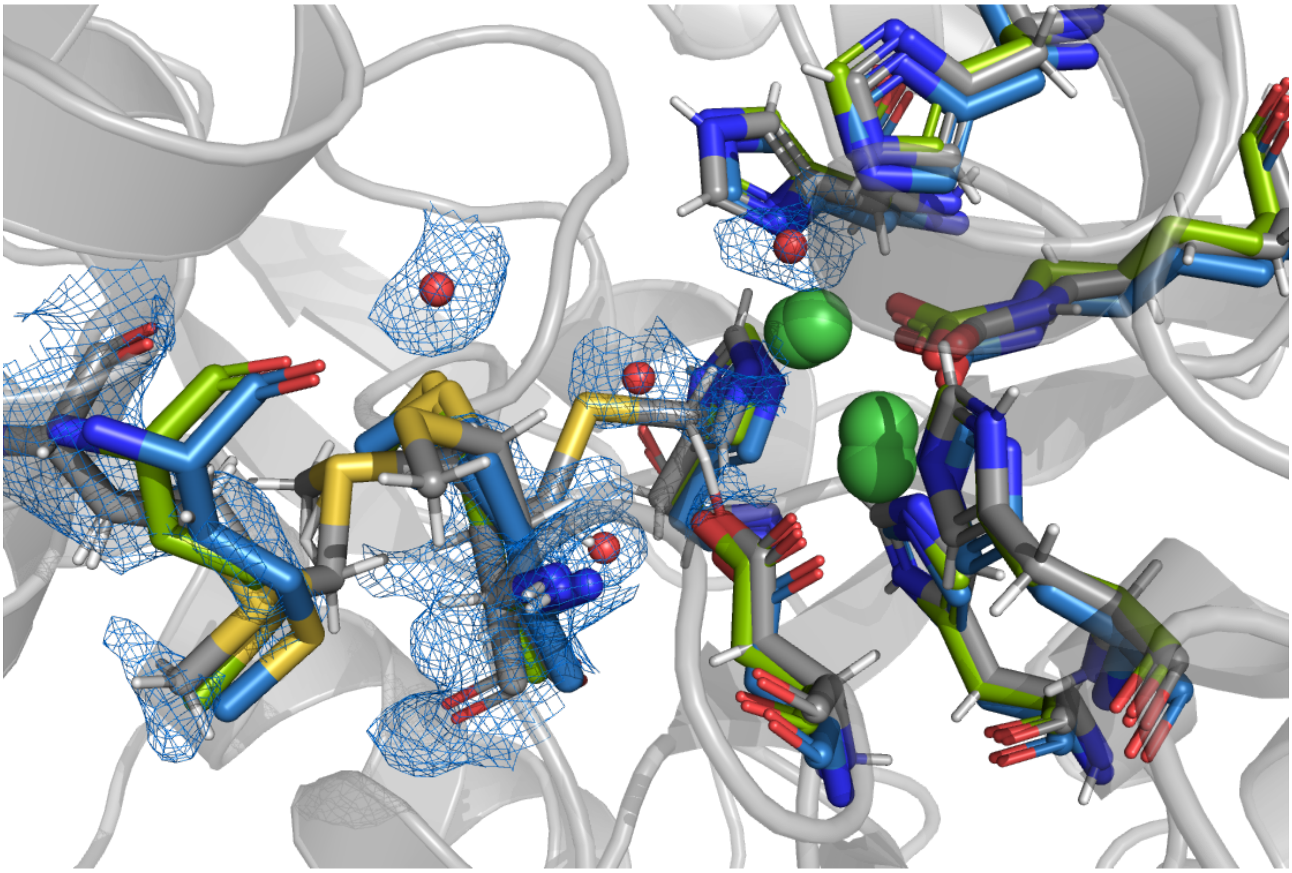

Supplementary Figure 9 Met369 can adopt different conformations in the absence of the substrate or an inhibitor. *Y. enterocolitica* urease (grey), *S. pasteurii* urease (blue), *K. aerogenes* urease (green). One conformation could potentially reach the active site. There is no described function for this amino acid in the catalysis. The conformation close to the active site is only possible because the pocket is empty, which can be seen with an overlay of the active site from *K. aerogenes* urease (PDB: 1EJW) at 1.9 Å and *S. pasteurii* urease (PDB: 5OL4) at 1.28 Å resolution, respectively **(Figure 5d,e).**

Supplementary Tables

Supplementary Table 1 Cryo-EM data collection and image processing summary.

	Single shot	Multi shot
**Data collection**		
Microscope	Titan Krios	Titan Krios
Voltage [kV]	300	300
Direct electron detector	Gatan K2	Gatan K2
Zero-loss energy filter	GIF (20 eV slit width)	GIF (20 eV slit width)
Physical pixel size [Å]	0.639	0.639
Super-resolution mode	No	No
Total exposure [e^-/Å^2]	42	42
Exposure time [s]	8	8
Number of frames [per movie]	40	40
Movies acquired	2,252	2,243
Beam-image shift?	No	3 shots per hole
**Image processing (before merging)**		
Movies processed	2,115	2,197
Pixel size [Å]	0.639	0.639
Box size [pixels^2]	512	512
Particles picked (Gautomatch w/ templates)	87,204	107,399
Particles after 2D classification	87,032	80,239
Particles after 3D classification	51,173	62,884
Unmasked resolution [Å] (FSC 0.143)	2.52	2.39
Masked resolution [Å] (FSC 0.143)	2.20	2.10
**Image processing (after merging)**		
Particles in final reconstruction		119,020
Unmasked resolution [Å] (FSC 0.143)		2.20
Masked resolution [Å] (FSC 0.143)		1.98
Angular distribution efficiency (0 to 1)		0.78

Supplementary Table 2 Beam tilt estimation after merging the two datasets.

	**Multi-shot #1**	**Multi-shot #2**	**Multi-shot #3**	**Single-shot**
**Beamtilt x *(mrad)***	0.008	-0.218	-0.028	-0.078
**Beamtilt y *(mrad)***	-0.091	-0.127	-0.000	-0.065

Supplementary Table 3 Bayesian polishing training results.

	Multi Shot (before merging)	Single Shot (before merging)	Merged	Multi Shot #1	Multi Shot #2	Multi Shot #3	Single shot
**Number of particles in training** ***(approximate)***	5,000	5,000	10,000	10,000	10,000	10,000	10,000
**Sigma for Velocity (Å/e^-)** ***(smaller = shorter tracks)***	0.531	0.819	0.779	0.810	0.693	0.636	0.681
**Sigma for Divergence (Å)** ***(higher = more homogeneous tracks across micrograph, i.e. "rigid block")***	3,600	10,455	10,620	10,260	7,350	5,220	8,355
**Sigma for Acceleration (Å/e^-)** ***(smaller = straighter tracks)***	2.175	3.735	1.620	1.485	1.425	1.500	0.795

Supplementary Table 4 Sequence identity between different ureases and *Y. enterocolitica* urease.

**Organism**	**PDB code**	**Nr. Of Genes**	**Stoichiometry α-(β)-(γ)**	**ureA (%)**	**ureB (%)**	**ureC (%)**
S. pasteurii	5O4L	3	3-3-3	60.6	46.7	57.5
K. aerogenes	1EJW	3	3-3-3	60.6	52.9	58.7
H. pylori	1E9Z	2	12-12	52.0	50.4	57.6

Supplementary Table 5 RMSD values between each chain of our *Y. enterocolitica* urease model versus structures deposited at the PDB. Values are given in Ångstroms. Values in parenthesis indicate the number of Cα atom pairs matched with and without pruning. The program matchmaker from UCSF Chimera was used to calculate sequence alignments and RMSD values between each chain in respective models.

	**Chain A**	**Chain B**	**Chain C**	**Active site**
**1E9Z**	0.860/0.942 (98/100)	0.702/4.424 (107/124)	0.642/1.248 (537/561)	0.634
**Alignment Score**	339.8	376.8	1874.2	-
**5OL4**	0.706/0.706 (100/100)	0.756/2.999 (108/120)	0.673/1.171 (542/563)	0.293
**Alignment Score**	369.3	327.5	2002.7	-
**1EJW**	0.607/0.607 (100/100)	0.622/1.054 (97/101)	0.611/0.916 (538/559)	0.270
**Alignment Score**	637.2	269.4	1957.0	-

Supplementary Note 1

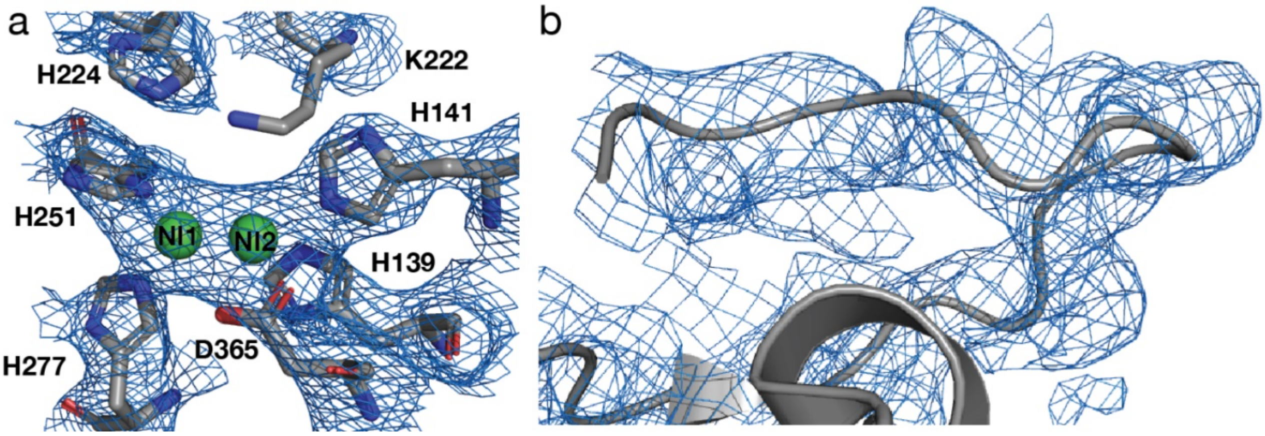

Supplementary Figure 10 Low resolution crystal structure of *Y. enterocolitica* urease.

Structure determination by X-ray crystallography yielded a 3.01 Å resolution density, which was sufficient to see the higher oligomeric state of *Y. enterocolitica* urease, but not for detailed analysis of the assembly mechanism and active site. Comparison of the model based on the high-resolution cryo-EM data *vs.* the previous crystal structure shows a few differences. The previous X-ray model did not include residue 100 of ureA and residues 31-33, 148-162 of ureB. Residues 148-162 form a helix, which was previously not observable. This helix sits right at the interface between three ureB proteins and constitutes the interaction between the trimers.

Supplementary Methods

For X-ray crystallographic analysis of the *Y. enterocolitica* urease, *Y. enterocolitica* was differently purified than described in the **Methods** for cryo-EM. The protein was precipitated using 40-60% w/v ammonium sulfate, resuspended and dialyzed in 0.15M NaCl, 50mM Tris pH 8.0. It was further purified using a 45 ml self-packed DEAE FF XK26/20 (Sigma, DFF100) and a Superdex 200 10/300 GL column. The SEC was also used for buffer exchange to 20 mM HEPES, 100 mM NaCl pH 7. Urease crystals grew at 20°C at 10 mg/ml in 0.1M CHES pH 9.5; 50% (v/v) PEG 200. Urease crystals belonged to space group H32 with unit cell parameters of a= 157.2 Å, b = 157.2 Å and c= 774.6 Å, with four molecules per asymmetric unit. The structure was determined by molecular replacement with PHASER [3] using the urease crystal structure from *Klebsiella aerogenes* (PDB: 1FWB) [4]. Model building and structure refinement were performed with Coot [5] and Buster-TNT [6]. The atomic coordinates for this model have been deposited in the Protein Data Bank under the accession code 4Z42.

Supplementary References

1. Chen, S. *et al.* High-resolution noise substitution to measure overfitting and validate resolution in 3D structure determination by single particle electron cryomicroscopy. *Ultramicroscopy* **135**, 24–35 (2013).

2. Rosenthal, P. B. & Henderson, R. Optimal Determination of Particle Orientation, Absolute Hand, and Contrast Loss in Single-particle Electron Cryomicroscopy. *J. Mol. Biol.* **333**, 721–745 (2003).

3. McCoy, A. J. *et al.* Phaser crystallographic software. *J. Appl. Crystallogr.* **40**, 658–674 (2007).

4. Pearson, M. A., Michel, L. O., Hausinger, R. P. & Karplus, P. A. Structures of Cys319 variants and acetohydroxamate-inhibited Klebsiella aerogenes urease. *Biochemistry* **36**, 8164–8172 (1997).

5. Emsley, P. & Cowtan, K. Coot: model-building tools for molecular graphics. *Acta Crystallogr. Sect. D* **60**, 2126–2132 (2004).

6. Blanc, E. *et al.* Refinement of severely incomplete structures with maximum likelihood in BUSTER-TNT. *Acta Crystallogr. Sect. D Biol. Crystallogr.* **60**, 2210–2221 (2004).
